# Approach using the bath preparation containing rice extract for severe pruritus in a patient receiving hemodialysis: A case report

**DOI:** 10.1002/ccr3.8638

**Published:** 2024-03-07

**Authors:** Hiroshi Suzuki, Katsuya Yamauchi, Shinya Uchida

**Affiliations:** ^1^ Yamauchi Pharmacy Shizuoka Japan; ^2^ Department of Pharmacy Practice & Science, School of Pharmaceutical Sciences University of Shizuoka Shizuoka Japan

**Keywords:** bath preparation, hemodialysis, moisturizers, pruritus, rice extract

## Abstract

Severe pruritus in a hemodialysis patient who had difficulty applying topical medication markedly reduced with the use of bath preparation containing rice extract. The bath preparation could be effective and an option for treating pruritus.

## 1 INTRODUCTION

Pruritus is a common complication that occurs in 40%–50% of patients receiving hemodialysis.^1^ This complication should not be disregarded because it decreases the quality of life, causes insomnia and depressive symptoms, and in severe cases, correlates with poor prognosis.^2^, ^3^, ^4^ Multiple factors, including the accumulation of uremic toxins, mineral bone disease, dialysis, dry skin, and abnormalities in itch control associated with endogenous opioids in the central nervous system, contribute to the development of pruritus in such patients.^5^, ^6^, ^7^ Therefore, treatment requires multiple approaches based on these factors.

An effective treatment algorithm for pruritus in patients undergoing hemodialysis has been reported.^8^ This algorithm recommends using moisturizers as first‐line treatment. However, pruritus in patients undergoing hemodialysis often involves the whole body, and applying topical drugs to the entire body is time‐consuming. In addition, many patients complain of itchy backs, and reaching their backs is difficult; spreading the moisturizer sufficiently across the entire target area is challenging. Consequently, some patients do not obtain the expected effect even when moisturizers are prescribed according to the above algorithm.

Chinese herbal bath therapy is a well‐known complementary and alternative medicine for uremic pruritus.^9^, ^10^ These therapies alleviate pruritus by accelerating blood circulation and promoting sweating. In bath therapy, patients can allow the active ingredients to be distributed throughout the body by soaking themselves in hot water with a bath preparation containing active ingredients. If bath preparations containing moisturizing ingredients are utilized in bath therapy, the moisturizing ingredients are expected to be sufficiently distributed to the affected areas and contribute to improving pruritus. Herein, we describe a clinical case of a hemodialysis patient in whom the continued use of a bath preparation containing moisturizing ingredients resulted in sustained suppression, followed by disappearance of severe pruritus.

## 2 CASE HISTORY/EXAMINATION

A 48‐year‐old man undergoing hemodialysis had had pruritus for over 3 years since his first visit to our pharmacy. As treatment for pruritus, he regularly took fexofenadine (60 mg/day) and nalfurafine hydrochloride (5 μg/day). In addition, he used topical steroids and a mixture of diphenhydramine and l‐menthol (1:1), depending on the skin condition.

## 3 METHODS (DIFFERENTIAL DIAGNOSIS, INVESTIGATIONS AND TREATMENT)

**FIGURE 1:**
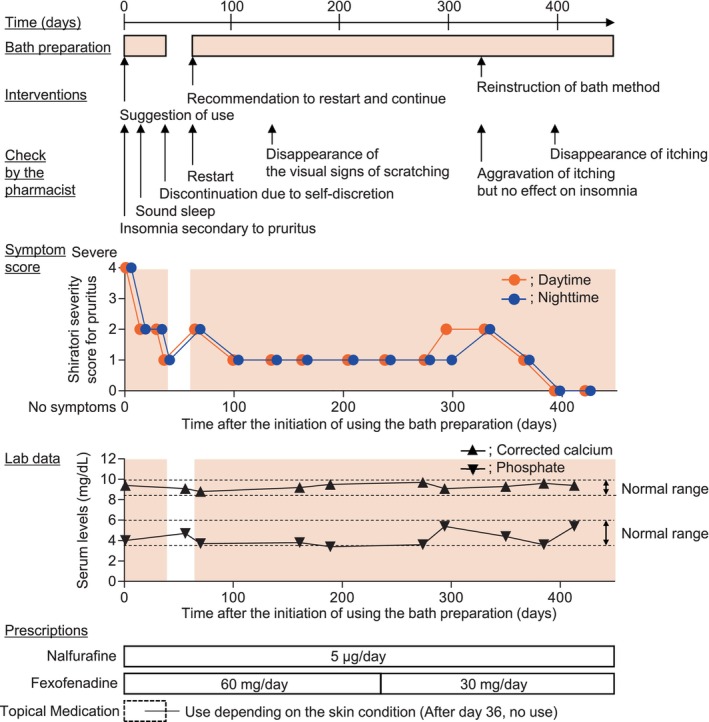
Clinical course of the patient. The Shiratori pruritus severity score indicates the intensity of itching on a 5‐point scale: 0, no symptoms; 1, very mild; 2, mild; 3, moderate; and 4, severe. The “Normal range” in the plot area indicates normal serum phosphate and corrected serum calcium ranges according to the chronic kidney disease–mineral and bone disorder guidelines (Japan). The boxes under the plot indicate the medication schedule.

On Day 0, the patient complained of insomnia secondary to severe itching. He had severe pruritus on his back and arms. He constantly scratched his arms during the medical interviews. The pharmacist had this patient assess his itching using the Shiratori severity score for pruritus.^11^ In Japan, the Shiratori severity score is routinely used in clinical practice.^8^, ^12^ Daytime pruritus was evaluated based on the following 5‐point scale: 0, No itching at all (no symptoms); 1, tolerable without scratching (very mild); 2, subsides after light scratching (mild); 3, subsides after considerable scratching; 4, does not subside with repeated scratching (severe). Nighttime pruritus was evaluated based on the following 5‐point scale: 0, no itching at all (no symptoms); 1, slight itching at bedtime, but not enough to consciously scratch (very mild); 2, subsides after light scratching; does not wake up due to itching (mild); 3, waking up due to itching; falling asleep after scratching once but unconsciously scratches while asleep (moderate); 4, hardly sleeps due to itching; constant scratching makes itchiness intense (severe). During the first assessment of itching at the pharmacy (Day 0), the patient's itching scores for daytime and nighttime itching were 4 each. The pharmacist suggested that the patient use a bath preparation containing rice extract (Barrier care®, Yushin Brewer Co., Ltd.) as a moisturizing ingredient. The pharmacist instructed the patient to add 30 mL of the bath preparation to 200 L of hot water in a home bathtub and bathe for 5–10 min on every non‐dialysis day. Additionally, he was instructed to avoid rubbing his body with a towel during washing. Subsequently, the pharmacist verbally confirmed the patient's bathing condition on a regular basis and had him evaluate his itching during daytime and nighttime, using the Shiratori severity score for pruritus. The transitions in these scores during the observational period are shown in Figure 1.

One week after the initiation of bath preparation (Day 7), the score values for both daytime and nighttime pruritus decreased from 4 to 2, and the patient slept well. Four weeks later (Day 35), the itching score decreased from 2 to 1, and topical medication was no longer necessary. As the patient's pruritus subsided, he thought that it had completely disappeared and, at his own discretion, discontinued the bath preparation.

On Day 63, 4 weeks after discontinuing the bath preparation, his itching worsened; the itching score values increased from 1 to 2 during daytime and nighttime. Owing to discontinuation of the bath preparation and worsening symptoms, the pharmacist recommended that the patient restart and continue using the bath preparation. The patient heeded the pharmacist's recommendations and restarted bath preparation, but not topical medication application. Five weeks after the restart (Day 98), itching score values decreased from 2 to 1 during daytime and nighttime. Five weeks later (Day 133), he did not scratch his arms during the medical interviews, and the visual signs of scratching on his arm disappeared. His itching remained subdued with continued use of the bath preparations, and the itching score values were maintained at 1 point during daytime and nighttime for 17 weeks.

On Day 252, the dose of fexofenadine was reduced from 60 to 30 mg/day, owing to stabilization of the pruritus. Three weeks after this decrease (Day 273), no worsening of itching was observed. Three weeks later (Day 294), the score value for daytime pruritus increased from 1 to 2; 5 weeks later (Day 329), the score value for nighttime pruritus increased from 1 to 2. However, the patient did not wake up owing to itching or restart the use of topical medications. Here, the pharmacist reinstated the bathing method instruction.

Five weeks after the reinstruction (Day 364), the patient's itching subsided, and its score values decreased from 2 to 1 at daytime and nighttime. Furthermore, on Day 392, his itching disappeared (daytime and nighttime pruritus score values decreased from 1 to 0), and this state was sustained for 4 weeks (Day 420).

On non‐dialysis days, the patient took a 10 min bath in the bath preparation. No adverse events associated with the bath preparation were observed throughout the observation period, and the patient was able to continue with the bath preparation. There was no change in the prescribed medications for pruritus, except for the reduction of fexofenadine dose. In addition, serum phosphate and corrected serum calcium levels were within the normal range (serum phosphate; 3.7–5.4 mg/dL, corrected serum calcium; 8.8–9.7 mg/dL) as per the chronic kidney disease–mineral and bone disorder (CKD‐MBD) guidelines in Japan^13^; there was no change in the prescribed medications for the control of those levels.

## 4 CONCLUSION AND RESULTS (OUTCOME AND FOLLOW‐UP)

Healthcare professionals should prescribe and dispense moisturizers, periodically confirm the patient's skin condition, and follow‐up on their use. Particularly in patients who have difficulty applying topical medication sufficiently to the affected areas, using bath preparations containing moisturizing ingredients may be effective and is expected to be applied as an option for treating pruritus.

## 5 DISCUSSION

Severe pruritus is associated with poor prognosis in patients receiving hemodialysis,^2^, ^3^ and therefore, a positive treatment approach is required for patients with pruritus. The patient described in this case report had severe hemodialysis‐associated pruritus. The patient had never been prescribed moisturizers, although the treatment algorithm for hemodialysis‐associated pruritus recommends the use of moisturizers as the first step.^8^ The patient had difficulty reaching his back to apply the moisturizer. In addition, the patient's mother, the only family member living with him, was cognitively impaired, and unable to support him. Therefore, instead of suggesting that physicians prescribe moisturizers, the pharmacist recommended using a bath preparation containing rice extract, which has a moisturizing effect on the skin. By using this bath preparation, the patient allowed the moisturizing ingredients to reach the affected area by soaking in hot water with the bath preparation. Consequently, the pruritus was expected to improve. Furthermore, because rice extract has a skin‐purifying effect, exfoliating dead skin cells,^14^ the body can be cleaned without scrubbing with a towel; therefore, the skin barrier function is maintained, improving pruritus. However, because the bath preparation is classified as an over‐the‐counter drug in Japan and its use is not covered by insurance, the patient had to bear the financial burden. After explaining this to the patient, the pharmacist suggested using the bath preparation and obtained his consent.

The patient's severe pruritus markedly reduced with the initiation of bath preparation. Although his itching worsened temporarily due to the cessation of bath preparation, it improved after restarting it based on the pharmacist's recommendation. Furthermore, continued bath preparation suppressed his itching symptoms throughout the observational period, except for a temporary worsening of itching after decreased fexofenadine dose. As described above, the patient's itching improved with the use of the bath preparation.

Only a few reports have shown the impact of moisturizing bath preparations containing ceramide on pruritus in patients receiving hemodialysis.^15^, ^16^ This report is the first to describe the effect of bath preparations containing rice extract on pruritus in such patients. Rice extract contains phenolic compounds, betaine, and squalene, which exhibit moisturizing and anti‐inflammatory effects.^17^, ^18^ These effects may improve skin moisture and physical stability, leading to pruritus inhibition, and skin improvement. In addition, we took advantage of the skin‐purifying effects of the rice extract and instructed the patient not to rub his body with a towel. This may have also contributed to the improvement in pruritus by maintaining skin barrier function. The pharmacist's support, including the bathing method instructions, reinstating the use of the bath preparation after discontinuation, and periodic checks of the patient's bathing condition, may have also contributed to the improvement observed in this case. In addition, the fact that the intervention was offered as a part of the patient's daily life (bathing) may have made it easier for the patient to accept and continue the treatment.

This was a case report, and because there was no control arm, the effect of the bath preparations on pruritus in patients undergoing hemodialysis could not be proven. However, the intensity of pruritis symptoms varied with the initiation and discontinuation of bath preparation. In addition, despite reports that chronic kidney disease associated pruritus does not spontaneously resolve,^19^, ^20^ the patient's severe pruritus symptoms significantly improved with the use of the bath preparation. Thus, it is believed that introducing bath preparation facilitated the improvement of pruritus. Elevated serum phosphate and corrected serum calcium levels are also considered factors affecting pruritus in patients receiving hemodialysis.^3^ However, in this case, both levels were within the normal range as per the Japanese CKD‐MBD guidelines throughout the observational period.^13^ Finally, this approach may not be acceptable for patients who do not have a habit of bathing and should be introduced with caution.

## AUTHOR CONTRIBUTIONS

**Hiroshi Suzuki:** Conceptualization; investigation; methodology; writing – original draft; writing – review and editing. **Katsuya Yamauchi:** Supervision. **Shinya Uchida:** Conceptualization; project administration; writing – original draft; writing – review and editing.

## FUNDING INFORMATION

This study did not receive any specific grants from funding agencies in the public, commercial, or non‐profit sectors.

## CONFLICT OF INTEREST STATEMENT

The authors have no conflicts of interest to declare.

## ETHICS STATEMENT

This article was written in compliance with the Helsinki Declaration.

## CONSENT

Written informed consent was obtained from the patient to publish the details of his medical case in accordance with the journal's patient consent policy.

## Data Availability

The data supporting this case report's findings are included in this article. Further inquiries can be directed to the corresponding authors.
